# Reciprocal longitudinal associations between job insecurity and depressive symptoms in young working women: the SLOSH study 2010–2020

**DOI:** 10.1007/s00420-026-02230-6

**Published:** 2026-08-19

**Authors:** Ylva Lindberg, Kristiina Rajaleid, Göran Kecklund, Paraskevi Peristera, Anna Nyberg

**Affiliations:** 1 Department of Public Health and Caring Sciences, Uppsala University, Uppsala, Sweden; 2 Department of Psychology, Stockholm University, Stockholm, Sweden; 3 Department of Public Health Science, Stockholm University, Stockholm, Sweden

**Keywords:** Depression, Gender, Labour market industry, Work environment, Multilevel structural equation modelling

## Abstract

**Objective:**

To investigate job insecurity and symptoms of depression in different labour market groups, with a specific focus on young women.

**Methods:**

Data were drawn from six waves of the Swedish Longitudinal Occupational Survey of Health (*n* = 14,827) in 2010–2020. Differences in job insecurity between young (≤ 35) working women and other gender and age (> 35) groups, and between labour market industries with different gender composition, were estimated. Longitudinal multilevel structural equation models were fitted to estimate reciprocal associations between job insecurity and depressive symptoms two years later, while adjusting for demographic and employment factors.

**Results:**

Younger workers reported significantly higher job insecurity compared with older. For older workers job insecurity was significantly lower in female dominated industries, whereas this pattern was not observed among younger workers. There was a medium strong path between job insecurity and depressive symptoms in young working women (0.106, *p* = 0.001), and men (0.111, *p* = 0.011). The reverse associations were not statistically significant. For older women and men, the effect sizes for associations between job insecurity and depression were small (0.044, *p* = 0.000 and 0.047, *p* = 0.000, respectively) whereas reverse paths estimates were of medium strength (0.077, *p* = 0.000 and 0.109, *p* = 0.000).

**Conclusion:**

The results indicated a clear age-related pattern. Higher levels of job insecurity and a stronger association between job insecurity and depressive symptoms were found among younger compared with older workers. No gender differences were observed. The results highlight the importance of addressing job insecurity as a concern in early working life.

**Supplementary Information:**

The online version contains supplementary material available at https://doi.org/10.1007/s00420-026-02230-6.

## Background

### Depression in young working women

In Sweden, common mental disorders, including depression, are the primary cause of long-term sickness absence among both women and men (Swedish Social Insurance Agency 2023). Over recent decades, the increase in mental health complaints has been particularly pronounced among younger cohorts, with young women most strongly affected (Public Health Agency of Sweden 2018). The reasons for this increase remain unclear (MIND 2018), although labour market conditions are likely to play a role (Public Health Agency of Sweden 2018).

Research consistently documents gender-based disparities in working conditions and career opportunities (Campos-Serna et al. 2013). Men and women often cluster in different industries with distinct working conditions (Nyberg et al. 2021), which may contribute to gendered health risks during the first years in the labour market. Yet, little is known about how specific stressors affect young workers, particularly young women, and how working conditions and the broader work environment influence their mental health (Law et al. 2020; van Veen et al. 2023). One recent qualitative study found that young workers explained the connection between psychosocial work factors and their mental health in somewhat different ways than older workers, with job insecurity emerging as a key factor (van Veen et al. 2024).

### Job insecurity and depression in young working women

Global competition and technological advances have contributed to the growth of non-standard forms of employment in recent decades (Kreshpaj et al. 2020). Fixed-term contracts are the most common type and are associated with greater long-term insecurity. Compared with permanent employees, those on temporary contracts report higher levels of perceived job insecurity, poorer working conditions, and lower pay (Hünefeld and Köper 2016). Job insecurity is defined as the subjective fear of losing one’s job (De Witte 1999; De Witte et al. 2016), a perception that is not necessarily based on objective threats (De Witte 1999; Låstad et al. 2016). When referring to *job insecurity* in the current study, the subjective construct is intended. Although fixed-term contracts are more prevalent among young workers and women (Statistics Sweden 2024a), findings on whether the experience of job insecurity differs by gender or age remain inconclusive (Låstad et al. 2016).

In general working populations, job insecurity is associated with depression (Blackmore et al. 2007; Högnäs et al. 2022; Magnusson Hanson et al. 2015; Wang et al. 2012), suicide mortality (Blomqvist et al. 2022), and psychotropic drug use (Blomqvist et al. 2020). However, a systematic review of psychosocial working conditions and mental health among workers up to age 35 identified only one relatively old study on depression and job insecurity, measured at the occupational level (van Veen et al. 2023; Zimmerman et al. 2004). Thus, very little is known about the specific relationship between job insecurity and depressive symptoms among young workers, particularly young women. To date, no study has explicitly examined this association with a gender perspective in younger age groups.

In the present study, early working life was operationalised as age 35 years or younger. In Sweden, labour market establishment is often delayed due to prolonged education and other transitions before stable employment is attained. The age threshold was therefore chosen to capture a period characterised by labour market establishment, career development, and family formation, all of which may increase vulnerability to employment insecurity. This life phase frequently coincides with childbearing and increasing caregiving responsibilities, potentially exacerbating financial strain and challenges of work–family balance. Prolonged insecurity during these years may therefore increase the risk of depressive symptoms. In addition, younger women generally have limited accumulated work experience and insufficient economic and social resources with which to buffer the consequences of insecure employment, while still facing persistent gender gaps in pay and career opportunities (Statistics Sweden 2024b). These combined pressures suggest that job insecurity may be a particularly relevant psychosocial stressor among young women and therefore warrants specific investigation.

Furthermore, while job insecurity has been linked to depression in general working populations, the reverse association between depression and job insecurity is less researched and understood (De Witte et al. 2016; Högnäs et al. 2022). Still, ongoing depressive symptoms may heighten perceptions of job insecurity through negative interpretive biases and reduced work functioning, creating the potential for a reciprocal cycle between insecurity and depression. From a theoretical perspective, job insecurity can be understood within Conservation of Resources (COR) theory (Hobfoll and Ford 2007), which proposes that stress arises when valued resources are threatened or lost. Employment represents an important economic and social resource (Lindberg et al. 2025, 2026), and perceived threats to continued employment may therefore contribute to psychological strain and depressive symptoms. Conversely, depressive symptoms may reduce personal resources and coping capacity, potentially increasing perceptions of insecurity. COR theory therefore provides a useful framework for understanding both prospective and reciprocal associations between job insecurity and depressive symptoms.

In summary, previous longitudinal studies on job insecurity and mental health have primarily examined general working populations or focused on unidirectional associations. There is a lack of age- and gender-specific perspectives in the current literature. Incorporating these dimensions might help shed light on the uneven distribution of mental ill-health in the population. By applying age- and gender-stratified reciprocal models, the present study extends the literature by examining how associations between job insecurity and depressive symptoms vary across the working life course.

### Aims

The overall aim was to investigate job insecurity and symptoms of depression in different labour market groups, with a specific focus on young (≤ 35) working women.

The specific aims were:

1. To compare the distribution of job insecurity in different gender and age groups (≤ 35 vs. > 35) on the labour market, and furthermore to compare the distribution of job insecurity in labour market industries categorised by gender composition (female-dominated, male-dominated and gender-integrated) for men and women of both age groups.
2. To estimate the reciprocal longitudinal association between job insecurity and depressive symptoms in young working women and in other gender and age groups.

## Methods

### Study design

A longitudinal study, based on six waves of cohort data collected between 2010 and 2020.

### Study sample

**Fig. 1:**
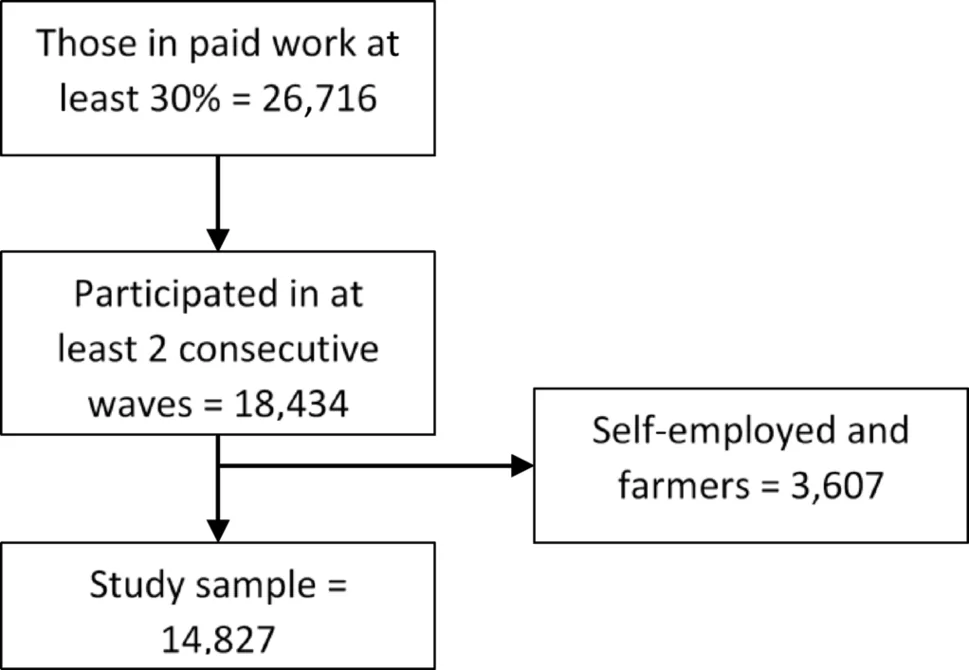
Flow chart of the sample selection process

Data were drawn from the Swedish Longitudinal Occupational Survey of Health (SLOSH), a cohort derived from a nationally representative sample of the Swedish working population. The cohort data include information on areas such as working conditions, social situation, and health and well-being. Data for the cohort were collected through questionnaires, conducted every other year between 2006 and 2022, and once per year thereafter. Survey data are linked to several administrative national registers (Magnusson Hanson et al. 2018; The Swedish Longitudinal Occupational Survey of Health). In the present study, data from the SLOSH waves from 2010 to 2020 (six waves) were included. The number of responses varied between waves, from 9880 to 20,316, and the response rate varied between 48 per cent and 57 per cent (The Swedish Longitudinal Occupational Survey of Health). Only those who were in paid work at least 30 per cent and had responded to the survey in at least two consecutive waves were included in the study sample (*n* = 14,827). Self-employed and farmers were excluded (Fig. 1). To assess potential selection bias related to the requirement of participation in at least two consecutive survey waves, characteristics of the final analytical sample were compared with those of the eligible working population before this restriction was applied (Supplementary Table S1). Differences were generally small, although younger women reported higher depressive symptoms and younger men somewhat higher job insecurity in the larger eligible sample .

### Variables

#### Predictor variable

Job insecurity was measured with three survey items asking participants about their current working situation: “Worry about dismissal”, “Worry about not keeping job”, and “Fear of job loss”. The items were measured on a five-point Likert scale ranging from 1 (completely disagree) to 5 (completely agree). In the analysis for the first aim, the three items were averaged to create an index, and in the analysis for the second aim, job insecurity was treated as a continuous latent variable. Cronbach’s α for job insecurity varied between 0.940 and 0.959 for the present sample, indicating excellent internal consistency.

#### Outcome variable

Symptoms of depression were measured with the SCL-6 depression scale (Magnusson Hanson et al. 2009, 2014). The scale contains six items asking participants to rate their experience over the past week regarding the extent of; “Lack of energy”, “Feeling blue/sad”, “Blaming yourself”, “Worrying too much”, “Lack of interest in things”, and “Everything is an effort”. The items were measured on a five-point Likert-scale ranging from 1 (not at all) to 5 (very much). The answers were recoded to a scale of 0–4 to create an index scale of 0–24. In the analyses, symptoms of depression was treated as a continuous latent variable. Cronbach’s α for symptoms of depression varied between 0.898 and 0.909, indicating good to excellent internal consistency.

#### Grouping variables

Gender was a dichotomous variable with the categories women and men. Age was measured in years and dichotomized into ≤ 35 years and 36 years and over. The industry variable was based on The Swedish Standard Industrial Classification (SNI, Statistics Sweden n.d.), which classifies work according to main occupational activity. The 20 SNI categories were grouped into three industry categories based on the proportion of women and men in the SNI category (Härenstam and Nyberg 2021). Categories with more than 60 per cent women were classified as female-dominated, those with between 40 and 60 per cent women and men were classified as gender-integrated, and categories with more than 60 per cent men were classified as male-dominated. The industry variable was used as a grouping variable in the analyses for Aim 1 and as a covariate in the analyses for Aim 2.

#### Covariates

Income was derived from register data of annual income in SEK and for the purpose of this study, was divided into deciles. Level of education was derived from registers and categorised into elementary school, high school, university < 3 years, and university ≥ 3 years. Marital status was measured with the question, “*Are you single or married/cohabiting?”* and children living at home with the question, “*Do you have any children living at home?”* Both were treated as dichotomous variables. Employment type was measured with the question, “*What type of employment do you have?”* with the response options permanent, project, temporary, hourly, and other. The answers were dichotomized into “permanent” (permanent) and “non-permanent” (project, temporary, hourly, other) employment. All covariates were specified as time-varying and measured at each survey wave. Consequently, the models adjusted for changes in income, education, marital status, children living at home, employment type, and industry gender composition over time.

### Analytical strategy

Because the assumption of normality did not hold, the non-parametric alternatives of Mann-Whitney U test for two groups and Kruskal-Wallis ANOVA for more than two groups were used to compare the distribution of job insecurity between groups. Data from the most recent included SLOSH wave (2020) were used. Since data collected during 2020 may have been influenced by the COVID-19 pandemic, a sensitivity analysis using data from the previous wave of data collection (2018) was performed. For the longitudinal analyses, autoregressive cross-lagged panel models (CLPM) within a multilevel structural equation modelling (SEM) framework were fitted to the data (SLOSH 2010–2020), separately for each gender and age group. Measurement occasions were modelled at the within-person level and individuals at the between-person level. This specification reduces the influence of stable between-person differences and facilitates the examination of within-person change over time. It furthermore allows the examination of reciprocal associations over time, while accounting for temporal stability in the constructs and concurrent associations between the variables at each time point (Newsom 2015). In the analysis, observation pairs with a time lag of two years were used, and one individual can thus contribute more than one observation pair. To assess stability over time in the measurement properties of the latent variables, longitudinal measurement invariance (Chen 2007; Mackinnon et al. 2022; Newsom 2015) was tested for job insecurity and symptoms of depression. This indicated no measurement variance over time. The fit indices Comparative Fit Index (CFI), Tucker Lewis Index (TLI), the Root Mean Square Error of Approximation (RMSEA), and the Standardized Root Mean Square Residual (SRMR) were used to evaluate model fit. Acceptable fit is assumed when CFI ≥ 0.90, TLI ≥ 0.90, RMSEA ≤ 0.08, and SRMR ≤ 0.08 (Little 2013). Standardised coefficients are reported. Cross-lagged effect sizes were interpreted according to benchmark values proposed by Orth et al. (2022), with 0.03 representing a small effect, 0.07 representing a medium effect, and 0.12 representing a large effect. Missing data were handled using Full Information Maximum Likelihood (FIML) under the assumption that data were Missing At Random (MAR). The MAR assumption was considered plausible because the SLOSH cohort contains rich information on demographic, socioeconomic, employment-related, and family characteristics that are associated with both study participation and the study variables. The analyses further adjusted for several time-varying covariates (employment type, industry gender composition, education, income, marital status, and children living at home), which are likely to explain much of the systematic missingness. Furthermore, item-level missingness was low (below 5% for all variables and below 1% for most variables). FIML estimates model parameters using all available observations rather than excluding participants with incomplete data, thereby reducing the loss of information associated with listwise deletion. SPSS version 30 was used for data management, descriptive statistics, and distribution comparisons, and Mplus version 8.10 was used for multilevel CLPM SEM analyses.

### Ethical considerations

The study was approved by the Swedish Ethical Review Authority, number 2022-00417-01, and performed in accordance with the Declaration of Helsinki.

## Results

### Descriptive statistics

The descriptive statistics are presented for the most recent wave (2020), stratified by gender and age group. As shown in Table 1, 90–95 per cent of the sample had a permanent position, about 80 per cent were married or cohabiting, and most women and younger men had a university education of 3 years or longer.

**Table 1:** Study variables presented by gender and age group, data SLOSH 2020 (*n* = 8319)

	Women ≤ 35	Men ≤ 35	Women > 35	Men > 35
**Sample ** ***n***	156	93	4763	3307
**Job insecurity**^a^ **mean (SD)**	1.48 (0.90)	1.58 (1.00)	1.31 (0.75)	1.38 (0.82)
**Symptoms of depression**^b^ **index mean (SD)**	6.67 (5.27)	6.68 (5.77)	5.60 (4.99)	4.45 (4.56)
**Industry n (%)**				
FD	56 (36.1)	11 (12.0)	2400 (50.9)	468 (14.4)
GI	76 (49.0)	52 (56.5)	1842 (39.1)	1528 (46.8)
MD	23 (14.8)	29 (31.5)	472 (10.0)	1267 (38.9)
**Employment type n (%)**				
Permanent	145 (95.4)	82 (90.1)	4404 (94.6)	2996 (95.4)
Non-permanent	7 (4.6)	9 (9.9)	253 (5.4)	143 (4.6)
**Median income tSEK**	352	439	409	490
**Education n (%)**				
Elementary school	2 (1.3)	0 (0)	133 (2.8)	185 (5.6)
High school	32 (20.6)	33 (35.5)	1608 (33.8)	1416 (42.8)
University < 3 years	11 (7.1)	4 (4.3)	301 (6.3)	370 (11.2)
University ≥ 3 years	110 (71.0)	56 (60.2)	2719 (57.1)	1336 (40.4)
**Marital status n (%)**				
Single	32 (20.5)	18 (19.4)	1072 (22.7)	614 (18.7)
Married/ cohabiting	124 (79.5)	75 (80.6)	3641 (77.3)	2670 (81.2)
**Children at home n (%)**				
Yes	91 (58.3)	50 (53.8)	1944 (41.6)	1446 (44.5)
No	65 (41.7)	43 (46.2)	2733 (58.4)	1803 (55.5)

a: 1–5, higher scores indicate more job insecurity; b: 0–24, higher scores indicate more symptoms of depression; SD, standard deviation; SLOSH, Swedish Longitudinal Occupational Survey of Health; FD, female dominated; GI, gender integrated; MD, male dominated

### Distribution of job insecurity by gender, age, and industry gender composition

#### Gender and age

There was no statistically significant difference in job insecurity between young working women and men (≤ 35 years; *p* = 0.157). However, younger women reported significantly higher job insecurity compared with women in the older age group (> 35 years; *p* = 0.004), and younger men reported higher job insecurity compared with older men (*p* = 0.003). A similar pattern was observed in the 2018 data, except that there was no statistically significant difference in job insecurity between younger and older men.

#### Industry gender composition

**Fig. 2:**
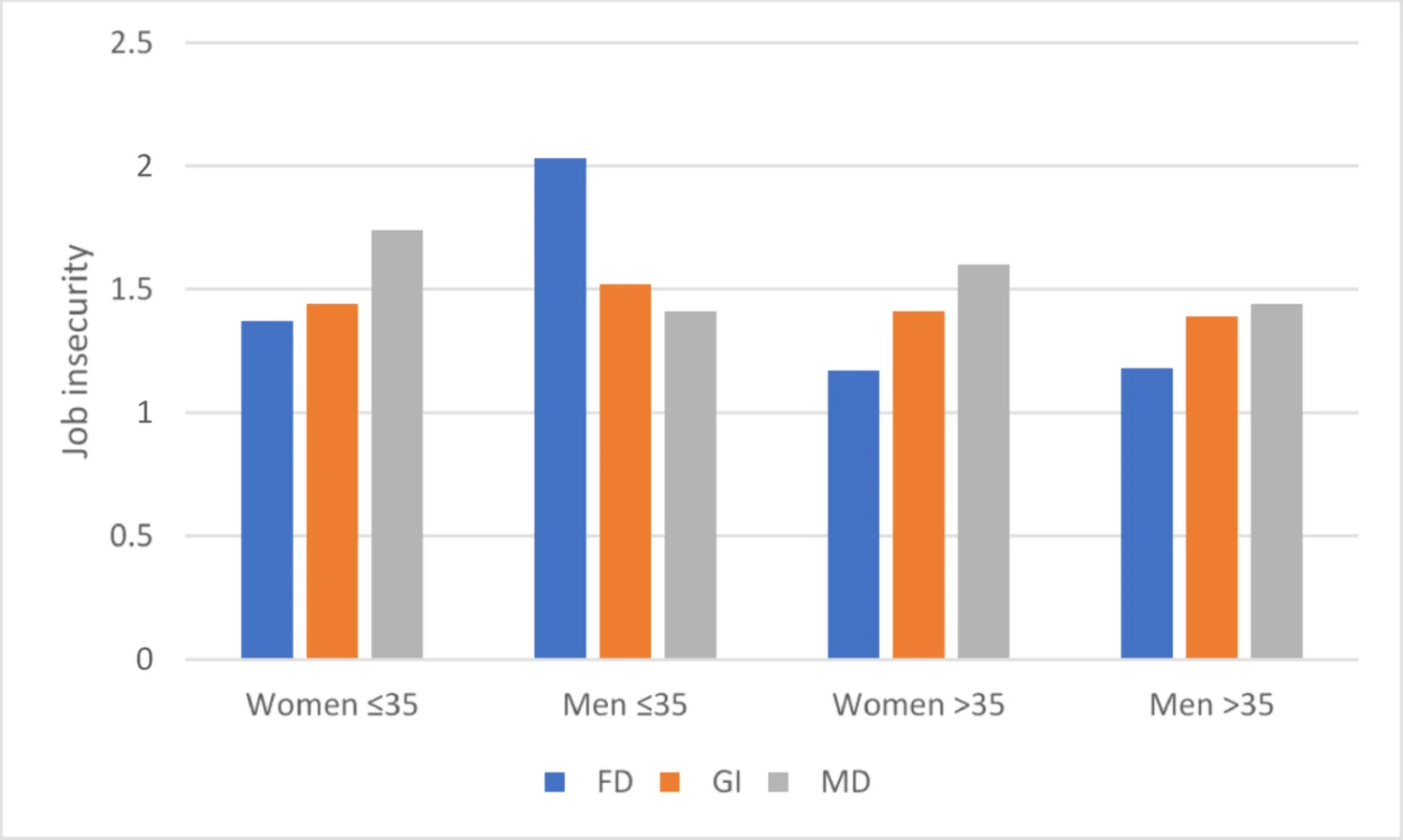
Job insecurity per industry category for each gender/age group, 2020 data. FD, Female dominated; GI, Gender integrated; MD, Male dominated

For younger women and men, no statistically significant differences in job insecurity were observed between female-dominated, gender-integrated, or male-dominated industries (*p* = 0.119 and *p* = 0.145, respectively). For older women, job insecurity differed significantly between all three industry categories (adjusted *p* = 0.000 for all differences), with the lowest levels in female-dominated industries and the highest levels in male-dominated industries. In older men, job insecurity was significantly higher in both gender-integrated (adjusted *p* = 0.000) and male-dominated industries (adjusted *p* = 0.000) compared with female-dominated industries. There was no statistically significant difference between gender-integrated and male-dominated industries. Figure 2 illustrates job insecurity per industry category for each group. Looking at the 2018 data, some differences were observed compared with the results for 2020. For younger women, job insecurity was significantly higher in gender-integrated than in female-dominated industries (adjusted *p* = 0.015). For older women, there was no difference between gender-integrated and male-dominated industries (adjusted *p* = 0.138).

### Association between job insecurity and depressive symptoms in young women

**Fig. 3:**
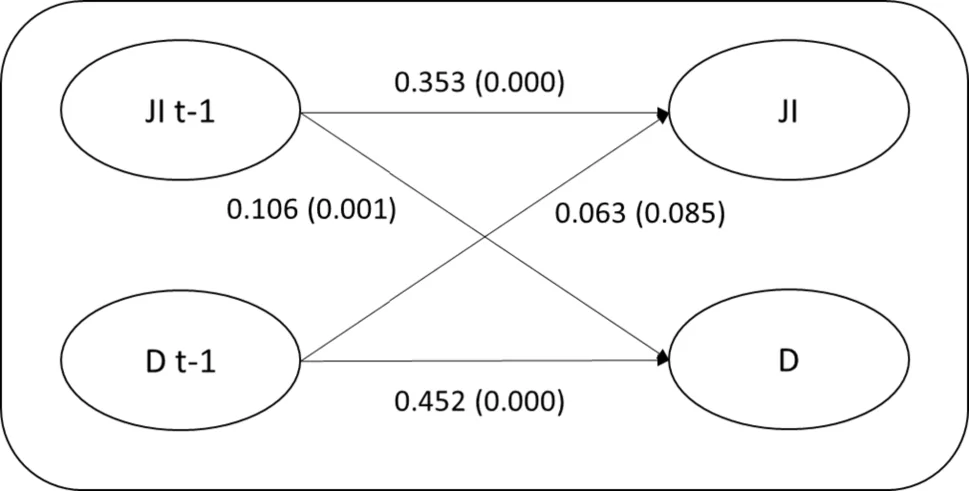
Bidirectional path estimates for associations between job insecurity (JI) and depressive symptoms (D) in younger women (*n* = 965), *P*-value in parentheses. Adjusted for employment type, industry gender composition, education, income, marital status, and children living at home. Standardized coefficients. t-1 indicates the first time point with two years between measurements

The sample included those who were in paid work at least 30 per cent and had responded to the survey in at least two consecutive waves between 2010 and 2020. Those who were self-employed or farmers were excluded. For the group women ≤ 35, the total number of individuals was 965. For this group, the longitudinal analysis showed a statistically significant association between job insecurity at the first time point and depressive symptoms at the second time point two years later (0.106, *p* = 0.001). According to benchmark values proposed by Orth et al. (2022) the strength of the effect size was interpreted as medium. The reverse association, from symptoms of depression to job insecurity, was not statistically significant (0.063, *p* = 0.085). Both autoregressive paths in the model showed statistically significant associations (Fig. 3; Table 2).

### Association between job insecurity and depressive symptoms in men and older women

**Fig. 4:**
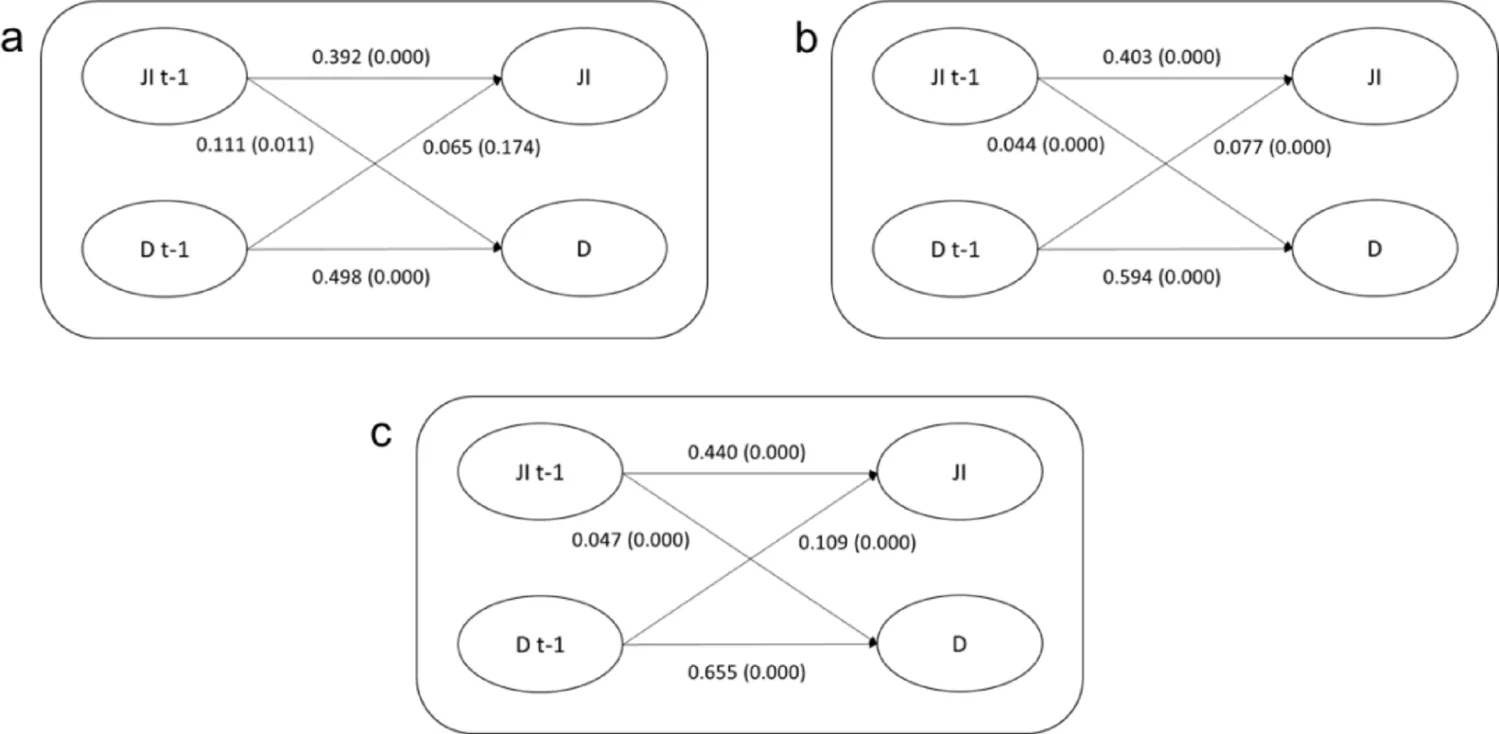
Bidirectional path estimates for associations between job insecurity (JI) and depressive symptoms (D) in (**a**) men ≤ 35 (*n* = 634),** b** women > 35 (*n* = 8264), and** c** men > 35 (*n* = 6024), *p*-value in parentheses. Adjusted for employment type, industry gender composition, education, income, marital status, and children living at home. Standardized coefficients. t-1 indicates the first time point with two years between measurements

The group of younger men included a total of 634 individuals. Results for this group (Fig. 4a; Table 2) showed a similar pattern to that observed among young women. Both autoregressive paths were statistically significant. There was a statistically significant association between job insecurity at the first time point and depressive symptoms at the second time point (0.111, *p* = 0.011). The reverse association was not statistically significant (0.065, *p* = 0.174). Consistent with the results for women ≤ 35, the effect size for the association between job insecurity and depressive symptoms was of medium strength for men ≤ 35 (Orth et al. 2022).

The groups of older women and men included a total of 8264 and 6024 individuals, respectively. Results for the older age groups differed from those of young women and men in that all paths in the model were statistically significant; the associations between job insecurity and depressive symptoms were therefore reciprocal. For women > 35, the path estimate between job insecurity at the first time point and symptoms of depression at the second time point was 0.044 (*p* = 0.000), and for men > 35 it was 0.047 (*p* = 0.000). The reverse path estimates were 0.077 (*p* = 0.000) for women and 0.109 (*p* = 0.000) for men (Fig. 4b and c; Table 2). The effect sizes for the paths between job insecurity at the first time point and depressive symptoms at the second time point were interpreted as small, while the effect sizes for the reverse paths were interpreted as medium for both groups (Orth et al. 2022).

**Table 2:** Standardised parameters (B), standard errors (SE), 95% confidence intervals (CI)

	Women ≤ 35 years		Men ≤ 35 years		Women > 35 years		Men > 35 years	
	B(SE)	95% CI	B(SE)	95% CI	B(SE)	95% CI	B(SE)	95% CI
**JI**								
JI t−1	0.353 (0.053)*	0.249; 0.456	0.392 (0.057)*	0.280; 0.503	0.403 (0.014)*	0.376; 0.430	0.440 (0.015)*	0.411; 0.469
D t−1	0.063 (0.036)	− 0.009; 0.134	0.065 (0.048)	− 0.029; 0.159	0.077 (0.008)*	0.061; 0.093	0.109 (0.010)*	0.090; 0.128
Employment type	0.308 (0.033)*	0.243; 0.372	0.256 (0.043)*	0.171; 0.340	0.133 (0.009)*	0.115; 0.152	0.081 (0.010)*	0.061; 0.101
Industry	0.084 (0.025)*	0.034; 0.133	0.022 (0.029)	− 0.036; 0.080	0.102 (0.007)*	0.089; 0.115	0.043 (0.007)*	0.029; 0.056
Education	− 0.060 (0.026)*	− 0.110; − 0.010	− 0.020 (0.030)	− 0.078; 0.039	− 0.042 (0.007)*	− 0.055; − 0.029	− 0.005 (0.008)	− 0.020; 0.010
Income	− 0.026 (0.023)	− 0.071; 0.018	− 0.018 (0.030)	− 0.078; 0.041	− 0.024 (0.007)*	− 0.037; − 0.011	− 0.025 (0.007)*	− 0.039; − 0.010
Marital status	− 0.023 (0.023)	− 0.069; 0.023	− 0.032 (0.032)	− 0.095; 0.031	− 0.030 (0.006)*	− 0.042; − 0.018	− 0.021 (0.007)*	− 0.035; − 0.006
Children at home	− 0.023 (0.023)	− 0.068; 0.022	0.001 (0.029)	− 0.056; 0.059	− 0.040 (0.006)*	− 0.051; − 0.029	− 0.040 (0.007)*	− 0.054; − 0.027
**D**								
JI t−1	0.106 (0.032)*	0.044; 0.168	0.111 (0.044)*	0.026; 0.197	0.044 (0.007)*	0.030; 0.058	0.047 (0.008)*	0.030; 0.064
D t−1	0.452 (0.038)*	0.378; 0.527	0.498 (0.056)*	0.388; 0.607	0.594 (0.008)*	0.578; 0.610	0.655 (0.010)*	0.636; 0.675
Employment type	− 0.014 (0.024)	− 0.061; 0.034	− 0.015 (0.032)	− 0.077; 0.047	− 0.021 (0.006)*	− 0.033; − 0.010	− 0.023 (0.006)*	− 0.035; − 0.011
Industry	0.026 (0.024)	− 0.021; 0.072	− 0.068 (0.029)*	− 0.125; − 0.011	0.022 (0.006)*	0.011; 0.034	0.001 (0.007)	− 0.013; 0.015
Education	− 0.056 (0.026)*	− 0.107; − 0.004	− 0.027 (0.030)	− 0.087; 0.032	0.034 (0.007)*	0.021; 0.047	0.032 (0.007)*	0.017; 0.046
Income	− 0.078 (0.023)*	− 0.123; − 0.034	− 0.014 (0.030)	− 0.073; 0.045	− 0.049 (0.006)*	− 0.062; − 0.037	− 0.049 (0.007)*	− 0.063; − 0.035
Marital status	− 0.103 (0.025)*	− 0.153; − 0.054	− 0.106 (0.034)*	− 0.174; − 0.039	− 0.035 (0.006)*	− 0.046; − 0.023	− 0.052 (0.007)*	− 0.066; − 0.037
Children at home	0.013 (0.023)	− 0.033; 0.059	0.041 (0.029)	− 0.015; 0.096	− 0.044 (0.006)*	− 0.055; − 0.032	− 0.032 (0.007)*	− 0.045; − 0.020

*: Significant on the 0.05 level. JI, Job Insecurity; D, Depressive symptoms

The models showed overall acceptable fit across age and gender groups. For women aged ≤ 35 years, women > 35 years, and men > 35 years, all fit indices met or were close to recommended thresholds. For men aged ≤ 35 years, model fit was slightly below recommended cut-offs for the CFI and TLI, while RMSEA and SRMR indicated acceptable fit (Table 3).

**Table 3:** Model fit job insecurity and symptoms of depression

	Women ≤ 35	Men ≤ 35	Women > 35	Men > 35
CFI	0.909	0.879	0.930	0.935
TLI	0.893	0.858	0.918	0.924
RMSEA	0.052	0.060	0.044	0.043
SRMR	0.064	0.068	0.042	0.043

CFI, Comparative Fit Index; TLI, Tucker Lewis Index; RMSEA, the Root Mean Square Error of Approximation; SRMR, the Standardized Root Mean Square Residual

## Discussion

The aim of the study was to investigate the distribution of job insecurity and its longitudinal reciprocal association with depressive symptoms among younger and older working women and men. Based on the study rationale, we expected that young working women may be particularly vulnerable to job insecurity. However, contrary to our expectations, age rather than gender appeared to be the more important dimension shaping both exposure to job insecurity and its longitudinal association with depressive symptoms. Younger workers reported more job insecurity than older workers, but there were no statistically significant differences between young women and men. Among younger workers, there was a medium strong longitudinal relationship between job insecurity and depressive symptoms. Among older workers, on the other hand, the association between job insecurity and depressive symptoms was reciprocal, with the association between depressive symptoms and later job insecurity being stronger (medium) than that between job insecurity and later depressive symptoms (small).

### Distribution of job insecurity in young women and other labour market groups

Younger women reported significantly higher job insecurity compared with older women, a result consistent with some earlier studies but inconsistent with others (Låstad et al. 2016). A suggested connection in younger workers experiencing more job insecurity is higher levels of unemployment (Låstad et al. 2016). This might be a contributing factor since there was an ongoing rise in unemployment in the year 2020 in Sweden (Statistics Sweden 2025). Another factor found in earlier studies (Låstad et al. 2016) is the type of contract that the employee has, that is, a permanent or temporary position. In general, younger groups show higher proportions of temporary positions (Statistics Sweden 2024a). In the present sample, however, the proportions of each employment type were similar between groups.

No significant gender difference was found within the younger cohort, but among older workers, on the other hand, job insecurity varied between genders. For the older workers, job insecurity also varied between labour market industries based on gender composition, with female-dominated sectors having the lowest levels. This suggests that female-dominated sectors provide a degree of security that benefits workers later in their career, possibly as a result of public-sector employment protections typical in Sweden. Younger women, however, may not yet have accumulated the tenure, networks, or specific skills required to benefit from such stability. They may thereby face the disadvantages of the adverse work environments specific to the educational and health and social care sectors (Cerdas et al. 2019) without receiving the potential buffering effects associated with more regulated employment conditions traditionally found in female-dominated sectors. This lack of protection for younger women compared with the older may, during a critical life phase characterised by household formation, parenthood, and early career establishment, contribute to the elevated risks of mental health problems observed in this group.

### Association between job insecurity and symptoms of depression in younger workers

In young workers of both genders, job insecurity was associated with later depressive symptoms with a medium effect size. This result is consistent with a substantial body of longitudinal research in general working populations (De Witte et al. 2016; Magnusson Hanson et al. 2015). However, few previous studies have specifically focused on younger workers, who often have weaker economic buffers, less experience navigating organisational structures, and limited bargaining power when negotiating working conditions. In addition, younger women may simultaneously face labour market discrimination and gendered expectations related to unpaid care (Statistics Sweden 2024b), amplifying the psychological burden of insecurity. An unexpected finding in that light was the absence of gender differences in the longitudinal associations between job insecurity and depressive symptoms in younger workers. Although young women have substantially higher rates of depressive symptoms at the population level, our results suggest that mental health outcomes may be similar for young women and men once insecurity is experienced. This may indicate that gender differences in mental health arise from a broader constellation of social, occupational, and life-course factors rather than from differential vulnerability to job insecurity itself. The absence of a reverse association in this group, meaning that depressive symptoms did not predict later job insecurity, may indicate that depressive symptoms in younger workers may be less influential with respect to work performance than in older groups, limiting their impact on perceived job security.

### Association between job insecurity and symptoms of depression in older workers

Among older women and men, all cross-lagged paths between job insecurity and depressive symptoms were significant, indicating a reciprocal relationship. However, the association between job insecurity and later depressive symptoms was small, whereas the reverse path from depressive symptoms to later insecurity was medium strong. It was not within the scope of the present study to identify underlying mechanisms, and we can only speculate about factors that may contribute to this pattern. For example, older workers experiencing depressive symptoms may to a greater extent than younger workers have reduced work functioning, lower productivity, or increased sickness absence, potentially heightening perceptions of insecurity and vulnerability in the workplace. Furthermore, employers may perceive older workers with health problems as less adaptable or costly to retain, especially in restructuring contexts, leading to subtle signals of insecurity that reinforce workers’ perceptions. Another possible explanation is that older workers may fear displacement more acutely because re-employment prospects decline with age. In such a context, depressive symptoms may amplify the subjective threat of job loss. Similarly, the mechanisms underlying the weaker association between job insecurity and depressive symptoms among older workers found in the present study require further exploration. One possible mechanism could be labour market selection. Individuals who are particularly sensitive to insecurity, or whose mental health is strongly affected by it, may exit high-insecurity environments earlier in their careers, leaving a more resilient group in older cohorts. This “healthy worker effect” could partially explain the age-graded differences observed.

Although the distinction between small and medium effect sizes should not be overinterpreted, the results indicate a clear age-related difference in the strength of the association. The longitudinal association between job insecurity and subsequent depressive symptoms was more than twice as large among younger workers compared with older workers. This suggests that job insecurity may represent a more salient psychosocial stressor during early working life, when financial resources, labour market position, occupational experience, and future career prospects are less established. Given that job insecurity is a relatively common exposure among younger workers, differences of this magnitude may also be relevant from a public health perspective. From a COR (Hobfoll and Ford 2007) perspective, the stronger longitudinal association between job insecurity and later depressive symptoms among younger workers may reflect their comparatively limited resource reserves. Early working life is often characterised by labour market establishment, career development, and family formation, while economic buffers, occupational experience, and professional networks are still being accumulated. Consequently, threats to continued employment may be experienced as particularly consequential. The absence of a significant reverse association among younger workers further suggests that resource threat, rather than resource depletion resulting from depressive symptoms, may be a more important factor underlying the observed relationship in this age group. In contrast, the reciprocal associations observed among older workers may be consistent with COR theory’s notion of loss spirals, whereby initial resource loss increases susceptibility to further losses over time. Depressive symptoms may reduce personal resources such as energy, self-efficacy, and coping capacity, thereby increasing perceptions of job insecurity, while insecurity itself may further erode psychological well-being. The stronger path from depressive symptoms to later job insecurity among older workers may therefore reflect a process in which health-related resource depletion becomes increasingly important later in the working life course.

### Strengths and limitations

A major strength of this study is the use of data from the Swedish Longitudinal Occupational Survey of Health (SLOSH), a large population-based cohort with repeated measurements over a ten-year period. The longitudinal design, combined with multilevel structural equation modelling, enabled the examination of temporal ordering and reciprocal associations between job insecurity and depressive symptoms, while accounting for autoregressive stability and concurrent associations. The use of validated multi-item measures, testing of longitudinal measurement invariance, and adjustment for a broad range of sociodemographic and employment-related covariates further strengthen the internal validity of the findings.

Several limitations should also be acknowledged. Although SLOSH is nationally based, the cohort includes individuals who were gainfully employed at baseline. Workers in the most precarious labour market positions, as well as those who exited employment due to severe mental health problems, may therefore be underrepresented. This likely results in conservative estimates of both job insecurity and depressive symptoms. Additionally, although differences between the eligible and analytical samples were generally modest, depressive symptoms among younger women and job insecurity among younger men were somewhat lower in the final sample. This suggests possible healthy-worker selection. Job insecurity and depressive symptoms were furthermore assessed using self-report measures, which may be subject to reporting bias.

Age was dichotomised into ≤ 35 and > 35 years in line with the study aim of examining job insecurity and depressive symptoms in early working life. However, dichotomisation may have reduced statistical power and obscured more gradual age-related differences.

The multilevel longitudinal design accounts for stable between-person differences and focuses primarily on within-person change over time. Even so, residual confounding from unmeasured time-varying factors cannot be ruled out. Examples include major life events, organisational changes, or other concurrent stressors that may influence both perceived job insecurity and depressive symptoms.

The multilevel cross-lagged panel modelling approach allowed us to examine reciprocal longitudinal associations while accounting for stable individual differences. However, the model does not provide the same explicit decomposition of within-person and between-person variance as random-intercept cross-lagged panel models (RI-CLPM). Future studies applying RI-CLPM could therefore provide additional insight into the robustness of the observed associations.

It should furthermore be noted that young men participating in SLOSH may constitute a selected group. In our analyses, depressive symptom levels among young men did not differ from those among young women, which contrasts with patterns typically observed in population-based studies. This potential selection bias may have attenuated expected gender differences in the present analyses. The younger age groups were smaller than the older groups, which may have reduced statistical power and contributed to less optimal model fit in some analyses, particularly among younger men. The findings for younger men should therefore be interpreted with some caution, as model fit was weaker in this group than in the other age and gender strata. Although RMSEA and SRMR indicated acceptable fit, both CFI and TLI fell below recommended thresholds, suggesting greater uncertainty regarding the parameter estimates.

Although the cross-sectional comparisons were based on data collected during 2020, sensitivity analyses using pre-pandemic data from 2018 showed largely similar age-related patterns, supporting the robustness of the findings.

Taken together, by combining a longitudinal national cohort with age- and gender-stratified analyses of cross-lagged within-individual change over time, this study provides novel evidence highlighting the importance of examining psychosocial work environment exposures in early working life as distinct from later career stages.

## Conclusions

This study demonstrates clear age-related differences in both the distribution of job insecurity and its longitudinal association with depressive symptoms. Younger workers report higher levels of job insecurity than older workers, and a stronger longitudinal association between job insecurity and depressive symptoms. There were no gender differences. In contrast, among older workers, the association was reciprocal, with depressive symptoms more strongly predicting subsequent job insecurity than the reverse.

These findings suggest that job insecurity constitutes a particularly salient psychosocial work stressor in early working life, whereas health-related selection processes may become more prominent later in workers’ careers. From an occupational health perspective, the results underscore the importance of addressing job insecurity early, before reciprocal cycles between mental health and perceived insecurity are established. Interventions and policies aimed at improving employment stability and psychosocial working conditions for young workers may therefore play an important role in preventing mental ill-health across the working life course.

Although the observed effect sizes were small to medium according to established benchmarks, they may still be of substantial public health relevance. Job insecurity is a common exposure in working populations, and even modest associations can translate into a considerable burden of depressive symptoms when large groups are exposed. From an occupational health perspective, reducing job insecurity among young workers could therefore contribute to improvements in population mental health.

### Practical implications

Practical measures may include increasing employment stability, improving communication during organisational change, strengthening supervisory support, and ensuring access to occupational health services for young workers. Policymakers may also consider labour market initiatives aimed at reducing prolonged employment insecurity during the transition from education to stable employment.

## Supplementary Information

Below is the link to the electronic supplementary material.

Supplementary Material 1

## Data Availability

Given restrictions from the ethical review board and considering that sensitive personal data are involved, the data that support the findings of this study are not possible to make freely available. Access to the data may be provided to other researchers in line with Swedish law and after consultation with the Stockholm University legal department. Requests for data stored at the Psychobiology and Epidemiology Institute, Department of Psychology, Stockholm University, should be sent to data@slosh.se.
