# Supplementary material for: Reciprocal longitudinal associations between job insecurity and depressive symptoms in young working women: the SLOSH study 2010–2020
Source: Int Arch Occup Environ Health. 2026 Aug 19;99(7):41. doi: 10.1007/s00420-026-02230-6 (PMC13489989; doi:10.1007/s00420-026-02230-6)
Supplement: Supplementary file 1 [file 420_2026_2230_MOESM1_ESM.docx]

Table S1 Study variables presented by gender and age group, data SLOSH 2020, before including only those who had answered at least two consecutive surveys

|  | Women ≤35 | Men ≤35 | Women >35 | Men >35 |
| --- | --- | --- | --- | --- |
| **Sample** n | 260 | 143 | 5059 | 4031 |
| **Job insecurity^a^** mean (SD) | 1.53 (0.98) | 1.70 (1.13) | 1.32 (0.77) | 1.39 (0.82) |
| **Symptoms of depression^b^** index mean (SD) | 7.30 (5.60) | 6.74 (5.74) | 5.76 (5.11) | 4.49 (4.60) |
| **Industry** n (%) |  |  |  |  |
| FD | 96 (37.1) | 17 (12.0) | 2731 (50.1) | 552 (13.9) |
| GI | 124 (47.9) | 77 (54.2) | 2163 (39.7) | 1872 (47.1) |
| MD | 39 (15.1) | 48 (33.8) | 559 (10.3) | 1548 (39.0) |
| **Employment type** n (%) |  |  |  |  |
| Permanent | 231 (90.6) | 127 (90.1) | 5082 (94.3) | 3647 (94.9) |
| Non-permanent | 24 (9.4) | 14 (9.9) | 306 (5.7) | 197 (5.1) |
| **Median income** tSEK | 327 | 417 | 407 | 485 |
| **Education** n (%) |  |  |  |  |
| Elementary school | 2 (0.8) | 1 (0.7) | 152 (2.8) | 223 (5.5) |
| High school | 60 (23.3) | 54 (37.8) | 1890 (34.3) | 1755 (43.5) |
| University < 3 years | 18 (7.0) | 7 (4.9) | 349 (6.3) | 451 (11.2) |
| University ≥ 3 years | 178 (69.0) | 81 (56.6) | 3115 (56.6) | 1601 (39.7) |
|  |  |  |  |  |
| **Marital status** n (%) |  |  |  |  |
| Single | 48 (18.5) | 38 (26.8) | 1230 (22.6) | 736 (18.4) |
| Married/ cohabiting | 211 (81.5) | 104 (73.2) | 4220 (77.4) | 3261 (81.6) |
| **Children at home** n (%) |  |  |  |  |
| Yes | 155 (59.8) | 65 (46.1) | 2371 (43.9) | 1838 (46.5) |
| No | 104 (40.2) | 74 (53.9) | 3034 (56.1) | 2117 (53.5) |

a: 1–5, higher scores indicate more job insecurity; b: 0–24, higher scores indicate more symptoms of depression; SD: standard deviation; SLOSH: Swedish Longitudinal Occupational Survey of Health; FD: female dominated; GI: gender integrated; MD: male dominated.
